# High Expression of CD244 and SAP Regulated CD8^+^ T Cell Responses of Patients with HTLV-I Associated Neurologic Disease

**DOI:** 10.1371/journal.ppat.1000682

**Published:** 2009-12-04

**Authors:** Yoshimi Enose-Akahata, Eiji Matsuura, Unsong Oh, Steven Jacobson

**Affiliations:** Viral Immunology Section, Neuroimmunology Branch, National Institute of Neurological Disorders and Stroke, National Institutes of Health, Bethesda, Maryland, United States of America; Imperial College London, United Kingdom

## Abstract

HTLV-I-specific CD8^+^ T cells have been characterized with high frequencies in peripheral blood and cerebrospinal fluid and production of proinflammatory cytokines, which contribute to central nervous system inflammation in HTLV-I-associated myelopathy/tropical spastic paraparesis (HAM/TSP). However, little is known about the differences in CD8^+^ T cell activation status between asymptomatic carrier (ACs) and patients with HAM/TSP. The expression of CD244, a signaling lymphocyte activation molecule (SLAM) family receptor, was significantly higher on CD8^+^ T cells in HTLV-I-infected patients, both ACs and patients with HAM/TSP, than those on healthy normal donors (NDs). Blockade of CD244 inhibited degranulation and IFN-γ production in CD8^+^ T cells of patients with HAM/TSP, suggesting that CD244 is associated with effector functions of CD8^+^ T cells in patients with HAM/TSP. Moreover, SLAM-associated protein (SAP) was overexpressed in patients with HAM/TSP compared to ACs and NDs. SAP expression in Tax-specific CTLs was correlated in the HTLV-I proviral DNA loads and the frequency of the cells in HTLV-I-infected patients. SAP knockdown by siRNA also inhibited IFN-γ production in CD8^+^ T cells of patients with HAM/TSP. Thus, the CD244/SAP pathway was involved in the active regulation of CD8^+^ T cells of patients with HAM/TSP, and may play roles in promoting inflammatory neurological disease.

## Author Summary

Human T-lymphotropic virus type I (HTLV-I) is a retrovirus that persistently infects 20 million people worldwide. The majority of infected individuals are asymptomatic carriers of the virus, but 5–10% of infected people develop either adult T cell leukemia/lymphoma (ATL) or a chronic, progressive neurological disease termed HTLV-I-associated myelopathy/tropical spastic paraparesis (HAM/TSP). HAM/TSP is characterized by central nervous system (CNS) inflammation including HTLV-I-specific CD8^+^ T cells where disease progression and pathogenesis is associated with a dysregulation of antigen-specific CD8^+^ T cells, although the mechanism of this dysregulation remains to be defined. Here we demonstrate that a signaling lymphocyte activation molecule (SLAM) family of receptors, CD244, was overexpressed on CD8^+^ T cells of HTLV-I-infected patients than those of healthy normal donors, and that the upregulation of the adaptor protein, SAP, in CD8^+^ T cells distinguished HTLV-I infected individuals with and without neurologic disease. Both CD244 and SAP were associated with effector functions (high expression of IFN-γ) of CD8^+^ T cells in patients with HAM/TSP. This finding has important implication for T cell-mediated pathogenesis in human chronic viral infection associated with imbalance of immune function.

## Introduction

HTLV-I infects 20 million people worldwide [1]. While the majority of infected individuals are asymptomatic carriers (ACs) of the virus, 5–10% of infected people develop either adult T cell leukemia/lymphoma (ATL) [2] or a chronic, progressive neurological disease termed HTLV-I-associated myelopathy/tropical spastic paraparesis (HAM/TSP) [3],[4]. HAM/TSP is characterized by infiltration of perivascular inflammatory cells in the spinal cord including HTLV-I-specific CD8^+^ T cells CTLs [5],[6]. High frequencies of these effector cells have been demonstrated in peripheral blood with even higher frequencies in cerebrospinal fluid (CSF) of patients with HAM/TSP. HTLV-I-specific CTLs produce various factors including IFN-γ and TNF-α that may suppress viral replication and kill infected cells or promote bystander activation and killing of nearby resident glial cells [7]–[15]. These studies suggested that HTLV-specific CTLs might be immunopathogenic in the inflammatory lesions of patients with HAM/TSP.

Despite HTLV-I-specific CTL responses, HTLV-I proviral loads are significantly elevated in HAM/TSP patients compared to AC [16]. Increased expression particularly of the trans-activating viral gene encoding HTLV-I Tax has been suggested to play a role in HTLV-I disease progression [10],[17]. HTLV-I Tax induces the expression of a various cellular genes, including IL-2 [18], the α-chain of the IL-2 receptor (IL-2Rα) [19], IL-15 [20], and IL-15Rα [21]. Increased expressions of these critical immune mediators directly contributes to CD8^+^ T cell activation and the ex vivo T cell proliferation observed in patients with HAM/TSP [22]. Although HTLV-I-specific CTL responses have been demonstrated in ACs and patients with HAM/TSP [23],[24], high expression of IFN-γ in CD8^+^ T cells specifically in HAM/TSP patients compared to ACs have been reported to be induced by interaction with virus-infected CD4^+^ T cells and CD8^+^ T cells [8],[9],[25]. Recently, CD8^+^ T cells in patients with HAM/TSP, but not in ACs, were demonstrated to spontaneously degranulate and produce IFN-γ. Importantly, this CTL degranulation was shown to be mediated by HTLV-I infection of mononuclear phagocytes (MPs) with the concomitant expression of IL-15 [24]. Thus, the activation of HTLV-I-specific CTLs in HAM/TSP is associated with both virus and cytokines, although the relative contribution of each of these factors to the observed dysregulation of chronically activated virus-specific CD8^+^ T cells in patients with HAM/TSP remains to be determined.

Effector functions of CD8^+^ T cells are known to be regulated by various cellular receptors and their downstream molecules. The signaling lymphocyte activation molecule (SLAM) family of receptors and their associated adaptors play a pivotal role in the control of both innate and adaptive immunity [26]. Recent evidences indicate that the family of receptors and their signaling cascades are also involved in various inflammatory diseases, such as rheumatoid arthritis and inflammatory bowl disease [27]–[29]. SLAM family receptors consist of six immunoglobulin-like molecules named CD150 (SLAM), CD244 (2B4), CD229 (Ly-9), NK-T-B antigen (NTB-A; Ly-108), CD84 and CD2-like receptor activating cytotoxic T cells (CRACC) [26],[30]. These receptors are differentially expressed on various immune cell types. Most of these receptors recognize self-ligands, but only CD244 is implicated in heterotypic interactions with CD48. CD244 is present on natural killer (NK) cells, γδ T cells, activated CD8^+^ T cells, monocytes and basophils [31]–[37]. Importantly, the expression of CD244 on CD8^+^ T cells was correlated with T cell activation, CTL differentiation and exhaustion [31],[34],[35],[38],[39]. High CD244 expression on CD8^+^ T cells has been shown in patients with HIV-1 infection [40], acute infectious mononucleosis [41] and myelodysplastic syndrome [42]. Its ligand, CD48, is a glyco-phosphatidylinositol (GPI)-linked receptor broadly expressed on immune cells. CD244/CD48 interactions between antigen-specific CD8^+^ T cells and their targets or among CD8^+^ T cells themselves can augment cytotoxicity of their specific targets, IFN-γ production, and proliferation [43],[44]. These immune responses are regulated by downstream signals, including the adaptor molecule, SLAM-associated protein (SAP). SAP is a small SH2-domain containing protein, expressed in T cells, NK cells, NKT cells, and some B cells [45]–[47]. Following CD244/CD48 interaction, SAP binds with high affinity to the cytoplasmic tail of CD244 and regulates the signal transduction by recruiting SRC kinases [48]. Patients with X-linked lymphoproliferative syndrome (XLP) with SAP deficiency are characterized by a decrease in cytotoxic function of NK cells and EBV-specific CD8^+^ T cells [49], suggesting that SAP also has critical role in cytotoxic function of these cells. Since it has been demonstrated that activated HTLV-I specific CTL may play an important role in the pathogenesis of HAM/TSP and that CD244 is a crucial regulator of CTL function, we characterized the expression of CD244 on CD8^+^ T cells of HTLV-I-infected patients and examined their functions in patients with HAM/TSP. Here for the first time we demonstrate that CD244 was overexpressed on CD8^+^ T cells of HTLV-I-infected patients compared to healthy normal donors (NDs), and that the upregulation of the adaptor protein, SAP, in CD8^+^ T cells distinguished patients with HAM/TSP from ACs. Moreover, blocking of CD244 and knockdown of SAP by siRNA resulted in inhibition of CD8^+^ T cell function, degranulation and IFN-γ expression, of patients with HAM/TSP. These data suggest that CD244/SAP pathway is involved in active regulation of CTLs in patients with HAM/TSP.

## Results

### CD244 expression on CD8^+^ T cells of patients with HAM/TSP

**Figure 1:**
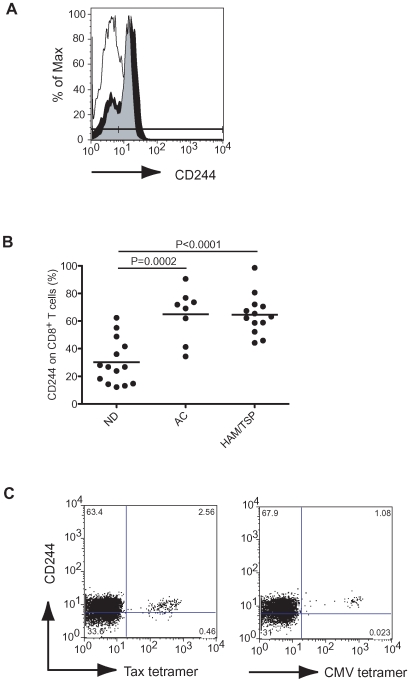
Expression of CD244 on CD8^+^ T cells of HTLV-I-infected patients. (A) Representative histograms of CD244 expression on CD8^+^ T cells of a ND, an AC and a patient with HAM/TSP. Staining in ND (opened histogram), AC (grayed histogram) and patient with HAM/TSP (closed histogram) were shown. (B) Comparison of CD244 expression in CD8^+^ T cells of NDs and HTLV-I-infected patients. The data were obtained from 14 NDs, 8 ACs and 13 patients with HAM/TSP. The horizontal line represents the median. (C) CD244 expression on antigen-specific CTLs. CD244 expressions were shown in Tax11-19- specific and CMVpp65-specific CD8^+^ T cells of a patient with HAM/TSP.

To determine whether CD244 expression correlates with activation of CD8^+^ T cells in HTLV-I-infected patients, CD244 expression on CD8^+^ T cells was examined by flow cytometry in NDs, ACs and patients with HAM/TSP. A representative histogram demonstrates that CD244 expression was higher on CD8^+^ T cells of HTLV-I-infected patients, both in an AC and a patient with HAM/TSP, than those of a ND (Figure 1 A). In NDs, group analysis demonstrated that 13–62% of CD8^+^ T cells expressed CD244 (n = 14; Figure 1 B). This result was comparable to previous results that reported approximately 30–50% of CD8^+^ T cells were CD244 positive in healthy donors [33]. In contrast, CD8^+^ T cells of HTLV-I-infected patients, both ACs and patients with HAM/TSP had significantly higher levels of CD244 expression; 35–90% and 45–98% respectively (Figure 1 B). There was no significant difference in CD244 expression between ACs and patients with HAM/TSP (P>0.05). Moreover, CD244 was expressed on Tax11-19-specific and CMV pp65-specific CD8^+^ T cells of HLA-A*0201 patient with HAM/TSP (Figure 1 C). NK cells of all subjects expressed CD244 (>95%), and did not show any differences in CD244 expression between NDs and HTLV-I-infected patients (data not shown). These results demonstrated that CD8^+^ T cells of HTLV-I-infected patients, including antigen-specific CTLs, showed significantly high CD244 expression, compared to NDs.

### Inhibition of degranulation and IFN-γ expression in CD8^+^ T cell by blockade of CD244

**Figure 2:**
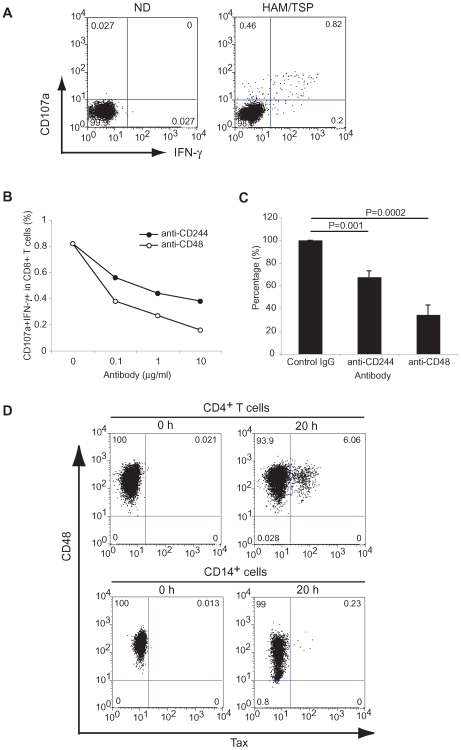
Involvement of CD244 in CD8^+^ T cell degranulation and IFN-γ expression of patients with HAM/TSP. (A) Representative dot plot of spontaneous degranulation and IFN-γ expression in CD8^+^ T cells of a ND and a patient with HAM/TSP after culture for 24 hours. The PBMCs were cultured for 24 hours without any exogenous stimulators. (B) Dose-dependent inhibition of spontaneous degranulation and IFN-γ expression in CD8^+^ T cells of a patient with HAM/TSP by anti-CD244 (closed circle) and anti-CD48 (opened circle). The PBMCs were cultured with each antibody for 24 hours. (C) Inhibitory effects of anti-CD244 and anti-CD48 on degranulation and IFN-γ production in CD8^+^ T cells of patients with HAM/TSP. The PBMCs were cultured with 1 µg/ml of control IgG, anti-CD244 or anti-CD48 for 24 hours. The amount of CD107a/IFN-γ expressions of CD8^+^ T cells cultured with control IgG were normalized to 100%, and then, those in PBMCs cultured with each antibody were calculated. The graph was prepared from data obtained from 7 patients with HAM/TSP. Error bars represent SD. (D) CD48 and Tax expression in HTLV-I-infected cells of a patient with HAM/TSP. The top panels show CD48 and Tax expression in CD4^+^ T cells before (left) and after (right) the culture for 20 hours. The bottom panels show CD48 and Tax expression in CD14^+^ cells before (left) and after (right) the culture for 20 hours.

As interactions of CD244 and its ligand, CD48, have been reported to augment cytotoxicity, IFN-γ production and proliferation of CD8^+^ T cells in mouse [43],[44], high expression of CD244 on CD8^+^ T cells may also augment functional human CD8^+^ T cell responses. It has been established that in *ex vivo* cultures of HAM/TSP PBMCs, CD8^+^ T cells are in close contact with HTLV-I-infected cells and rapidly function to kill these infected cells by secretion of lytic granules and cytokines [9],[12],[24],[25]. Therefore, to confirm the involvement of CD244 in this cytolytic process, we assessed CD8^+^ T cells of patients with HAM/TSP for their cytotoxic activity as defined by degranulation (CD107 expression) and IFN-γ expression by blocking CD244 or its ligand, CD48. Figure 2 A shows a representative dot plot of CD107a and IFN-γ expressions in CD8^+^ T cells of ND and patients with HAM/TSP after culture of whole PBMCs for 24 hours. As previously reported [24], CD8^+^ T cells of patients with HAM/TSP expressed both CD107a and IFN-γ after culture for 24 hours, whereas CD8^+^ T cells of ND did not (Figure 2 A). When anti-CD244 or anti-CD48 was titrated and cultured in PBMCs of a patient with HAM/TSP, both antibodies inhibited CD107a and IFN-γ expression in CD8^+^ T cells of a patient with HAM/TSP in a dose dependent manner (Figure 2 B). Figure 2 C shows the inhibitory effects of anti-CD244 and anti-CD48 (1µg/ml) on degranulation and IFN-γ expression of CD8^+^ T cells in patients with HAM/TSP (n = 7). Anti-CD244 significantly inhibited (32.5%) degranulation and IFN-γ expression in CD8^+^ T cells of patients with HAM/TSP compared with a control isotype IgG. Anti-CD48 had an even more pronounced inhibitory effect (65.5%). An additional established measure of HAM/TSP T cell activation *ex vivo* is the well-described observations of increased spontaneous T cell lymphoproliferation [50]. However, blockade of CD244 did not show any inhibitory effects on spontaneous lymphocyte proliferation of patients with HAM/TSP (data not shown). These results demonstrate that CD244/CD48 interactions might be specifically involved in cytotoxic CD8^+^ T cells dysregulation, especially degranulation and IFN-γ expression, of patients with HAM/TSP.

Although CD48 is broadly expressed on hematopoietic cells including lymphocytes and monocytes, viral infection such as HIV or EBV has been shown to decrease or increase CD48 expression on the infected cell, respectively [51],[52]. Therefore, we examined CD48 expression on CD4^+^ T cells and CD14^+^ cells, which are known to be *in vivo* reservoirs for HTLV-I in patients with HAM/TSP and gradually express HTLV-I Tax protein after short term *in vitro* culture [24],[53]. Figure 2 D shows a representative result of CD48 and HTLV-I Tax expression in CD4^+^ T cells and CD14^+^ cells of a patient with HAM/TSP. Both CD4^+^ T cells and CD14^+^ cells expressed CD48 before culture (Figure 2 B). CD48 expression on CD4^+^ T cells did not change over time despite the expression of HTLV-I Tax (Figure 2 B). This result supports previous studies that *in vitro* infection of HTLV-I in CD4^+^ T cells did not alter CD48 expression [54]. However, CD48 expression was partially downregulated in CD14^+^ cells after culture, but did not appear to be directly mediated by HTLV-I Tax expression, because Tax was not detected on the cells expressing low level of CD48 (Figure 2 B). Collectively, these results demonstrate that HTLV-I expression does not directly mediate CD48 expression on infected cells, suggesting that CD244-expressing HTLV-I-specific CTLs have the potential to interact with virus-infected CD48^+^ target cells.

### Distribution of CD244 during cell-cell interaction

**Figure 3:**
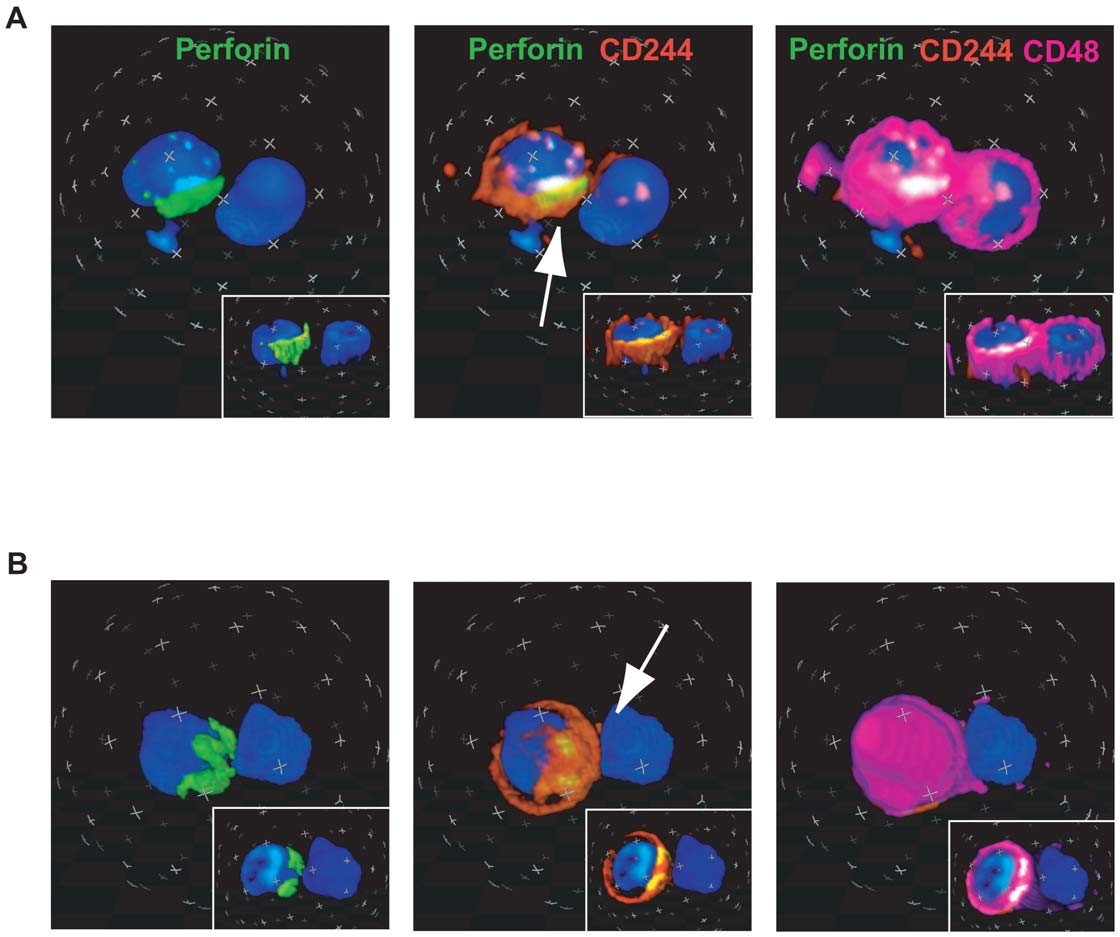
Distribution of CD244 in PBMCs of patients with HAM/TSP. After the culture for 8 hours, PBMCs were stained with antibodies against perforin, CD244 and CD48, and visualized through microscope. Two representative 3D images were shown in A and B. The image shows DAPI (blue), perforin (green), CD244 (orange) and CD48 (purple). In addition to DAPI and perforin (left), CD244 (middle) and CD48 (right) are merged. The white arrows indicate CD244 clustering at the cell contact area.

It is well known that interactions between effector CD8^+^ T cells and their targets establish a distinct immunological synapse organized by the accumulation of various immunoreceptors to cell-cell junctions associated with the polarization of lytic granules, such as perforin, leading to induction of cell death [55]. To support further the involvement of CD244/CD48 signaling on CD8^+^ T cells in HTLV-I-infected patients, the distribution of CD244 was visualized on cytotoxic lymphocytes (perforin^+^ cells) of patients with HAM/TSP after 8 hours *in vitro* culture when both perforin^+^ cells and polarizing perforin^+^ cells were most frequently visualized. As shown in Figure 3, immunofluorescence analysis demonstrated colocalization of CD244 on perforin^+^ cells (Figure 3 A and B). When these perforin^+^ cells were in contact with their targets, either strongly positive (Figure 3 A) or weakly positive (Figure 3 B) for CD48, polarization of perforin with the accumulation of CD244 was visualized at the cell-cell contact area (Figure 3 A and B, middle). The accumulation of CD244 on polarizing perforin^+^ cells was observed in more than 30% of all the polarizing perforin^+^ cells in contact with another cell. These results demonstrate that expression and subcellular distribution of CD244 and perforin on cytotoxic lymphocytes from HAM/TSP patients was translocated to the contact area (immunological synapse) with CD48^+^ target cells, suggesting that CD244 on cytotoxic lymphocytes is involved in recognition of target cells or activation of cytotoxic lymphocytes in HTLV-I-infected patients.

### SAP expression in CD8^+^ T cells of patients with HAM/TSP

**Figure 4:**
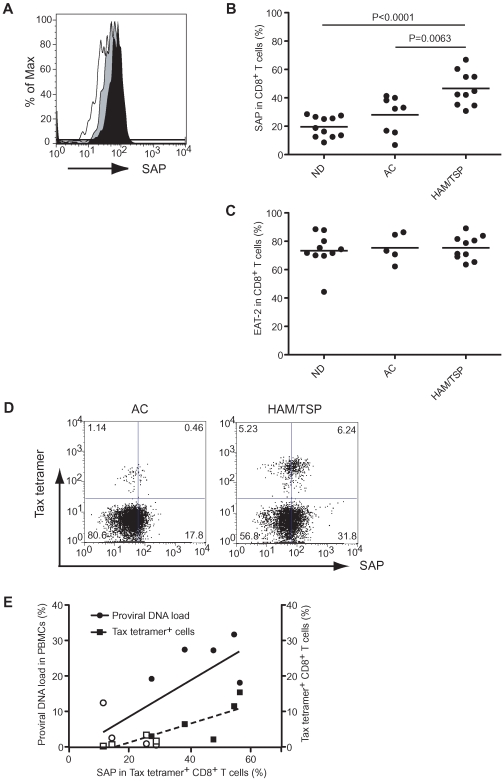
Expression of SLAM-associated proteins in CD8^+^ T cells of HTLV-I-infected patients. (A) Representative histograms of SAP expression in CD8^+^ T cells of a ND (opened histogram), an AC (grayed histogram), and a patient with HAM/TSP (closed histogram). (B) Comparison of SAP expression in CD8^+^ T cells of NDs (n = 11), ACs (n = 8) and patients with HAM/TSP (n = 10). The horizontal line represents the median. (C) Comparison of EAT-2 expression in CD8^+^ T cells of NDs (n = 10), ACs (n = 5) and patients with HAM/TSP (n = 10). (D) SAP expression in Tax11-19-specific CTLs of HTLV-I-infected patients. SAP expressions were shown in Tax11-19-specific CD8^+^ T cells of AC and patient with HAM/TSP. (E) Correlation of the HTLV-I-proviral DNA loads (circles; R^2^ = 0.4746, P = 0.0401) and the frequency of Tax11-19-specific CD8^+^ T cells (squares R^2^ = 0.6811, P = 0.0062) with SAP expression in the cells of AC (n = 4, opened circles and squares) and patients with HAM/TSP (n = 5, closed circles and squares).

Given the involvement of CD244/CD48 interaction on CD8^+^ T cells of patients with HAM/TSP, we asked how CD244/CD48 signaling might regulate CD8^+^ T cell function since high expression of CD244 was demonstrated on CD8^+^ T cells of both ACs and patients with HAM/TSP. It is known that NK cells constitutively express CD244 [33]. However, the regulation of these cells, either in a resting or activated state, is related to the expression of the SLAM-related adaptor proteins such as SAP and EAT-2, which play a role in controlling the active and inhibitory signal transduction in NK cells, respectively [32], [56]–[58]. To determine whether SLAM-related adaptor proteins were associated with active regulation of CD8^+^ T cells, the expression of the adaptor proteins, SAP and EAT-2, were compared in CD8^+^ T cells of NDs, ACs and patients with HAM/TSP. Representative results of SAP expression in CD8^+^ T cells are shown in Figure 4. SAP expression was higher in CD8^+^ T cells of a patient with HAM/TSP compared to that of ND and AC (Figure 4 A). Group analysis demonstrates that compared with the expression of SAP on CD8^+^ T cells of NDs and ACs, SAP expression was significantly increased in CD8^+^ T cells of patients with HAM/TSP (Figure 4 B). Although CD8^+^ T cells of ACs expressed SAP slightly higher than those of NDs, statistical analysis did not show any significant differences between these groups. The expression of SAP in NK cells were 43.9%, 44.6%, and 58.0% on average in NDs, ACs, and patients with HAM/TSP, respectively, and was not significantly different among groups. Likewise, EAT-2 was highly expressed in CD8^+^ T cells of both NDs and HTLV-I-infected individuals with no significant differences observed (Figure 4 C). These results demonstrate that CD8^+^ T cells of patients with HAM/TSP overexpress SAP compared to NDs and ACs, although EAT-2 expression was comparable in all three groups.

To assess SAP expression in antigen-specific CTL, we examined HTLV-I Tax11-19 tetramer^+^ CD8^+^ T cells in HLA-A*0201^+^ AC and patient with HAM/TSP. As shown in a representative histogram, SAP expression was higher in Tax tetramer^+^ CD8^+^ T cells of patient with HAM/TSP than the AC (Figure 4 D). To address SAP expression in these antigen-specific CD8^+^ T cells, we analyzed the HTLV-I proviral DNA loads in PBMCs and the frequency of Tax11-19 tetramer^+^ CD8^+^ T cells in HTLV-I-infected individuals as a function of SAP expression. As previously reported [9],[12], patients with HAM/TSP had higher frequencies of Tax11-19 tetramer^+^ CD8^+^ T cells, compared to ACs that correlated with HTLV-I proviral DNA loads. Figure 4E demonstrates that the amount of SAP expression in Tax11-19 tetramer^+^ CD8^+^ T cells was significantly correlated with HTLV-I proviral DNA loads in PBMCs (P = 0.0401, R^2^ = 0.4746) and the frequency of Tax11-19 tetramer^+^ CD8^+^ T cells in HTLV-I-infected individuals (P = 0.0062, R^2^ = 0.6811). These results suggest that expansion of HTLV-I-specific CD8^+^ T cells particularly in patients with HAM/TSP is associated with the expression of SAP and can distinguish patients with neurologic disease from HTLV-I infected asymptomatic carriers.

### Increase of SAP expression in IL-2- and IL-15-stimulated CD8^+^ T cells

**Figure 5:**
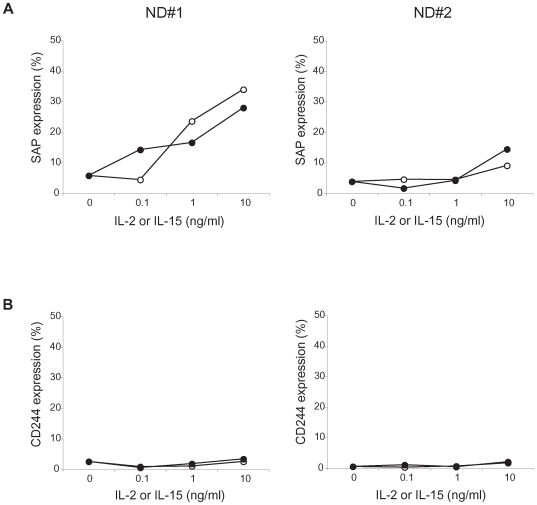
SAP and CD244 expressions in CD8^+^ T cells of NDs after stimulation with IL-2 or IL-15. (A) SAP expressions in CD8^+^ T cells isolated from ND PBMCs were compared after the culture with rhIL-2 (opened circle) or rhIL-15 (closed circle) for 7 days. The graphs were prepared from data obtained from two NDs (left and right). (B) CD244 expressions in CD8^+^ T cells isolated from ND PBMCs were compared after the culture with rhIL-2 (opened circle) or rhIL-15 (closed circle) for 7 days. The graphs were prepared from data obtained from two NDs (left and right).

HTLV-I Tax induces the expression of a various cytokine genes, including IL-2 and IL-15, which has been shown to be associated with CD8^+^ T cell activation and proliferation in patients with HAM/TSP [22]. IL-15 plays an important role in the prolonged maintenance of memory CD8^+^ T cell responses [59]. To examine whether IL-2 and IL-15 can upregulate SAP and CD244 expression in CD8^+^ T cells, purified CD8^+^ T cells from ND PBMCs (n = 2) were cultured with recombinant human IL-2 (rhIL-2) or rhIL-15 for 7 days, and expression of SAP and CD244 in these cells were analyzed. Both rhIL-2 and rhIL-15 stimulation induced SAP expression in CD8^+^ T cells of NDs, depending on the concentration of rhIL-2 or rhIL-15 (Figure 5 A). In addition, the magnitude of SAP expression in CD8^+^ T cells by IL-2 and IL-15 was variable among individuals. By contrast, CD244 expression on CD8^+^ T cells did not change after culture with rhIL-2 and rhIL-15 (Figure 5 B). Thus, IL-2 or IL-15 stimulated CD8^+^ T cells showed high expression of SAP, suggesting that the observed upregulation of SAP in CD8^+^ T cells of patients with HAM/TSP may be a function of increased cytokine production.

### Inhibition of degranulation and IFN-γ expression in CD8^+^ T cells by SAP knockdown

**Figure 6:**
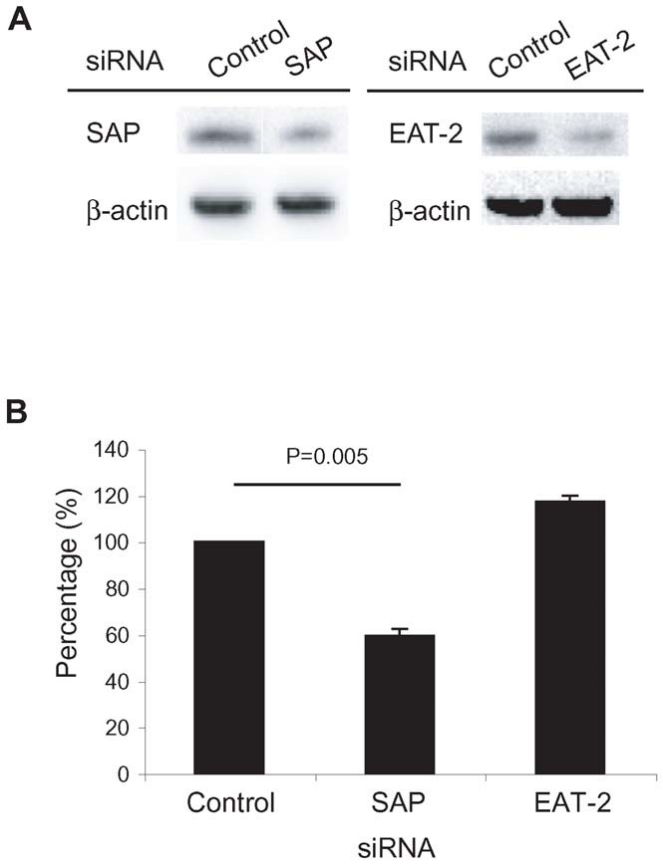
Inhibition of degranulation and IFN-γ expression in CD8^+^ T cells of patients with HAM/TSP by SAP siRNA. (A) SAP and EAT-2 expression in transfected CD8^+^ T cells with either control, SAP or EAT-2 siRNA was determined after the culture for 6 hours. The cell lysates were prepared from transfected CD8^+^ T cells, and each 10µg of the cell lysates was loaded on the gel. (B) Inhibitory effects of SAP or EAT-2 siRNA on degranulation and IFN-γ expression in transfected CD8^+^ T cells. Isolated CD8^+^ T cells from patients with HAM/TSP were transfected with control, SAP or EAT-2 siRNA, and then cocultured with autologous CD14^+^ cells. The amounts of CD107a/IFN-γ expressions in CD8^+^ T cells transfected with control siRNA were normalized to 100%, and then, those in CD8^+^ T cells transfected with SAP or EAT-2 siRNA were calculated. The graph was prepared from data obtained from three patients with HAM/TSP. Error bars represent SD.

Upregulation of SAP may be related to dysregulation of chronically activated CD8^+^ T cells in patients with HAM/TSP. To confirm the involvement of SAP on dysregulation of CD8^+^ T cells in patients with HAM/TSP, we examined degranulation and IFN-γ expression in CD8^+^ T cells of patients with HAM/TSP after knockdown of SAP or EAT-2 by siRNA. SAP and EAT-2 expression in transfected CD8^+^ T cells was determined after culture for 6 hours. Gene knockdowns by siRNA were effective against their respective targets at levels of 50–60% of baseline (Figure 6 A). After coculture with autologous CD14^+^ cells, CD8^+^ T cells transfected with SAP siRNA significantly decreased degranulation and IFN-γ expression (40.5% inhibition, P = 0.005) compared to CD8^+^ T cells transfected with control siRNA (Figure 6 B) while CD8^+^ T cells transfected with EAT-2 siRNA had no inhibitory effect. The inhibitory effect of SAP siRNA was similar to those of anti-CD244 on degranulation and IFN-γ expression in CD8^+^ T cells of patients with HAM/TSP (Figure 2 C). These results demonstrate that decreased SAP expression resulted in inhibition of degranulation and IFN-γ expression in CD8^+^ T cells of patients with HAM/TSP, supporting the role for SAP in the activation of cytotoxic CD8^+^ T cell function in patients with HAM/TSP.

## Discussion

Activation and dysregulation of CD8^+^ T cells in HTLV-I-infected patients have been suggested to be associated with disease progression and pathogenesis of HAM/TSP [7]–[15]. In this study, we have characterized that a member of the SLAM family of receptors, CD244, was overexpressed on CD8^+^ T cells of HTLV-I-infected patients compared to NDs, and demonstrated that the upregulation of the adaptor protein, SAP, in CD8^+^ T cells distinguished patients with HAM/TSP from ACs. The expression of CD244 on CD8^+^ T cells correlated with T cell activation [31],[34],[35],[38], as has been reported in patients with HIV-1 infection [40], acute infectious mononucleosis [41], and myelodysplastic syndrome [42]. CD244^+^ CD8^+^ T cells lack expression of CD45RA, CD62L, CD28, and CCR7 and acquire expression of perforin, granzyme B, and IFN-γ [31],[35],[38]. In addition, 2B4^+^ cells (also known as CD244) showed higher cytotoxicity than 2B4^−^ T cells [31],[34]. Since high frequency of CD45RA^−^CD27^+^ memory CD8^+^ T cells and high proliferation rate of CD8^+^ CD45RO^+^ T cells have been reported in patients with HAM/TSP [10],[60] and in HTLV-I-infected patients (both ACs and patients with HAM/TSP [61]), the expression of CD244 on CD8^+^ T cells in HTLV-I-infected patients is consistent with the interpretation that CD244 is a marker of memory CD8^+^ T cells and with persistent immune activation in HTLV-I-infected patients. High CD244 expression was demonstrated on HTLV-I Tax11-19-specific CD8^+^ T cells as well as CMV pp65-specific CD8^+^ T cells in a patient with HAM/TSP. Previous results have shown that CMV pp65-specific CD8^+^ T cells also showed high expression of CD244 whereas influenza virus-specific and melanoma antigen-specific CD8^+^ T cells showed low or negative expression of CD244, respectively [31]. Furthermore, high expression of CD244 has been recently reported to be related to CD8^+^ T cell exhaustion during chronic LCMV infection in mouse [39]. In our study, there were no differences in CD244 expression on CD8^+^ T cells between ACs and patients with HAM/TSP (Figure 1 B) and expression of CD244 in HTLV-I-infected patients did not show any direct correlation with HTLV-I infection such as HTLV-I proviral load or expression of Tax (data not shown). Thus, expression of CD244 on CD8^+^ T cells might indicate the activation state of CD8^+^ T cells or degree of CTL differentiation in chronic viral infection.

As suggested by the analyses of the disorder XLP, which is characterized by SAP deficiency and a decrease in cytotoxic function of NK cells and EBV-specific CD8^+^ T cells, the apparent activating effect of CD244 in human NK cells and CD8^+^ T cells may relate to preferential expression of SAP [32], [56], [62]–[64]. Here we have demonstrated a disease-specific upregulation of the adaptor protein, SAP, in CD8^+^ T cells of patients with HAM/TSP but not in NDs or ACs. This observation was specific for SAP since EAT-2 was comparably expressed in both NDs, and HTLV-I-infected patients and carriers. Moreover, the expression of SAP was higher in both total CD8^+^ T cells and in Tax11-19-specific CD8^+^ T cells in patients with HAM/TSP compared to ACs. Interestingly, while expression of CD244 in HTLV-I-infected patients did not show any correlation with HTLV-I infection, the upregulation of SAP in Tax11-19-specific CD8^+^ T cells was significantly correlated with the HTLV-I proviral DNA load in PBMCs and the frequency of Tax11-19-specific CD8^+^ T cells in HTLV-I-infected patients, suggesting that the expression of SAP is associated with the increase of proviral DNA and the expansion of antigen-specific CD8^+^ T cells. This is consistent with previous reports that demonstrated the relationship of disease progression with proviral DNA loads and virus-specific CD8^+^ T cells [17]. Upregulation of SAP was reported in splenocytes of mice infected with LCMV and MCMV [56], and in PBMCs of patients with infectious mononucleosis, even during early stages of the disease [41]. While SAP expression in NK cells is related to stimulation with NK cell activators, such as IL-2, IL-12, IFN-α and poly(I∶C) [56],[58], regulation of SAP expression in human CD8^+^ T cell is still unclear [64]–[66]. Since the expansion of HTLV-I-specific CD8^+^ T cells in HTLV-I infected patients is known to be influenced by various cytokines such as IL-2 and IL-15 as well as by virus antigen, we reasoned that these same stimuli would induce the expression of SAP that we have shown to be elevated in HTLV-I specific CD8^+^ T cells. In our study, stimulation of NDs CD8^+^ T cells with IL-2 and IL-15 induced SAP but not CD244 expression on CD8^+^ T cells. Indeed, while TCR activation was strongly correlated with expression of CD244 on CD8^+^ T cells, as previously reported [64], stimulation of cytokines such as IL-2 and IL-15 induced upregulation of SAP in CD8^+^ T cells. Therefore, compared to CD8^+^ T cells in ACs with high expression of CD244 and low expression of SAP, high expression of CD244 and SAP in CD8^+^ T cells of patients with HAM/TSP may be regulated by these cytokine-dependent activation as well as TCR dependent activation.

Results presented in this study also demonstrate that CD244/SAP pathway was functionally involved in cytotoxic activity of CD8^+^ T cells in patients with HAM/TSP. Immunofluorescence analysis visualized CD244^+^ perforin^+^ cytotoxic T cells in contact with CD48^+^ cells where clusters of CD244 and perforin were localized to the cell-cell contact area. These results demonstrated that CD244 on cytotoxic lymphocytes is involved in recognition of target cells or activation of cytotoxic lymphocytes. Recruitment of lytic molecules such as perforin to the cell-cell contact area suggests induction of target cell death. Moreover, analysis of CD8^+^ T cell function in patients with HAM/TSP as defined by degranulation and IFN-γ expression was inhibited by blockade of CD244 or knockdown of SAP, but not EAT-2. However, since spontaneous degranulation and IFN-γ expression was not detected in CD8^+^ T cells of ACs [24], we suggest that high expression of CD244 alone was not directly responsible for spontaneous degranulation and IFN-γ expression of CD8^+^ T cells. Rather, the overexpression of SAP in CD8^+^ T cells of patients with HAM/TSP, with high expression of CD244, might contribute to the CTL activity observed in this disorder by overproduction of IFN-γ and other inflammatory mediators. These results confirmed that expression of both CD244 and SAP are associated with CD8^+^ T cell activity, as previously reported [64]. The CD244/SAP pathway has been shown to regulate effector functions of NK and T cells depending on the expression level of CD244 and SAP [67]. Our results extends the significance of the CD244/SAP pathway in activation and regulation of virus-specific CD8^+^ T cell responses and has important implications for T cell-mediated pathogenesis of inflammatory neurologic disorders associated with an imbalance of immune function.

## Materials and Methods

### Patient samples

Blood samples were obtained from thirteen patients with HAM/TSP (HAM#1-13), eight AC (AC#1-8) and fourteen HTLV-I-seronegative healthy donors (ND#1-14). Diagnosis of HAM/TSP was based on World Health Organization (WHO) diagnostic criteria. Five patients with HAM/TSP and four AC were HLA typed as HLA-A*0201^+^. PBMCs were isolated by Ficoll-Hypaque (Lonza Walkersville, Walkersville, MD) centrifugation, and were cryopreserved in liquid nitrogen until use. Informed consent was written and obtained from each subject in accordance with the Declaration of Helsinki. The study was reviewed and approved by the National Institute of Neurological Disorders and Stroke Institutional Review Board.

### Antibodies and reagents

For flow cytometory, antibodies for human CD3, CD4, CD8, CD14, CD48, CD107a, CD244, IFN-γ, perforin (all from BD Biosciences, San Jose, CA), SAP (Cell Signaling, Danvers, MA), EAT-2 (Santa Cruz Biotechnology, Santa Cruz, CA), Tax 11–19/HLA-A0201 tetramer (provided by National Institute of Allergy and Infectious Disease MHC Tetramer Core Facility, Atlanta, GA) and CMV pp65/HLA-A0201 tetramer (Beckman Coulter, San Diego, CA) were used. Anti-Tax monoclonal antibody (Lt-4) was kindly provided by Dr. Y. Tanaka (University of the Ryukyus, Okinawa, Japan). For blocking experiments, both anti-CD244 and anti-CD48 were purchased from eBioscience (San Diego, CA). For immunofluorescence microscopy, primary antibodies used were anti-CD244 (R&D systems; Minneapolis, MN), anti-CD48 (AbD Serotec; Oxford, UK) and anti-perforin (BD Biosciences); and secondary antibodies used were Alexa 546 donkey anti-goat IgG (H&L), Alexa 647 goat anti-mouse IgG_1_, and Alexa 488 goat anti-mouse IgG_2b_ (all from Invitrogen; Carlsbad, CA), respectively. siRNAs specific for SAP mRNA, EAT-2 mRNA and control siRNA were purchased from Santa Cruz Biotechnology. Recombinant human (rh) IL-2 and rhIL-15 were purchased from Peprotech. (Rocky Hill, NJ).

### CD107a mobilization assay

CD107a mobilization assay was performed as previously described [24]. Briefly, PBMCs of ND or HTLV-I-infected patients were suspended in RPMI 1640 media supplemented with 10% FBS, 100 U/mL penicillin, 100 µg/mL streptomycin sulfate, and 2 mM L-glutamine, and cultured in 24 well plate in 5% CO_2_ incubator at 37°C for 24 hours. In blocking experiments, 0, 0.1, 1 or 10 µg/mL of each antibody (control IgG, anti-CD244 and anti-CD48) was added. Conjugated CD107a antibody, GoldiStop™ (BD Biosciences), and brefeldin A (Sigma, St. Louis, MO) were added into the culture for 5 hours before the time point for detection.

### Immunofluorescent staining and flow cytometry

Expressions of CD107a, IFN-γ, CD244, Tax, SAP or EAT-2 in the cultured or uncultured PBMCs were examined by flow cytometoric analysis. PBMCs were surface-stained with specific antibodies. In the case of combination staining with tetrameric complexes, PBMCs were stained with either Tax11-19 or CMV pp65-specific tetramer before surface staining. After being fixed and permeabilized with Fixation/Permeabilization solution (BD Biosciences), the cells were intracellular-stained with antibodies against IFN-γ, Tax, SAP or EAT-2 for each experiment. Flow cytometric analysis was performed using a FACSCalibur flow cytometer (BD Biosciences). The data were analyzed using FlowJo software (Tree Star, San Carlos, CA). To examine CD244 and SAP expression in CD8^+^ T cells stimulated with IL-2 or IL-15, CD8^+^ T cells were magnetically isolated from ND PBMCs by negative selection using CD8^+^ T cell isolation kit II (Miltenyi, Bergisch Gladbach, Germany) and cultured with appropriate concentration of rhIL-2 or rhIL-15 for 7 days.

### Immunofluorescence microscopy

Lab tech chamber glass slides (Nalge Nunc International, Rochester, NY) were coated with poly-L-lysine (Sigma) before use. PBMCs were plated into the chamber slides with RPMI media, and cultured for 8 hours. After washing with PBS including 1% FBS, the cells were stained with primary antibodies for CD244 and CD48 for 1 hour at room temperature. After fixation with 4% paraformardehide for 15 min and subsequent washing with PBS including 0.1% Triton-X, cells were stained with a primary antibody for perforin for 1 hour. After washing, each secondary antibody was applied, and DAPI was finally used for nuclear counterstaining. The stained cells were visualized with Zeiss 200M Axiovert inverted microscope equipped with mercury lamp house HBO-100 and four appropriate dichroic filters for DAPI and Alexa 488, 546 and 647 (Carl Zeiss MicroImaging Inc, Thornwood, NY). To evaluate the cell-cell contact and the distribution of perforin, CD244 and CD48, polarizing perforin^+^ cell and its contacting cell was first identified under the microscope and then the image picture was created using four filters for DAPI, perforin, CD244 and CD48. Three-dimensional (3-D) reconstructions of each section were assembled using Volocity 3-D imaging analysis software (Improvision, Waltham, MA). The image data was deconvoluted and modified into 3-D image, and the accumulation of CD244 on polarizing perforin^+^ cells in contact with the other cell was confirmed from 50 sets of the 3-D image data.

### HTLV-I proviral DNA load

DNA was extracted from total PBMCs of HLA-A*0201^+^ HTLV-I-infected patients using QIAamp DNA Blood Mini Kit (QIAGEN, Valencia, CA) and HTLV-1 proviral DNA load was measured using TaqMan system as previously described [68].

### Transfection with SAP small interfering RNA (siRNA)

CD8^+^ T cells and CD14^+^ cells were magnetically isolated from the patient's PBMCs by CD8^+^ T cell isolation kit II and CD14 MicroBeads (both from Miltenyi), respectively, according to the manufacturer's instructions. The purities of CD8^+^ T cells and CD14^+^ cells were confirmed as >90% cells and as approximately 95%, respectively. Purified CD8^+^ T cells (3×10^6^ cells) were transfected with 300 nM of control, SAP or EAT-2 siRNA using the Human T Cell Nucleofector Kit (AMAXA, Cologne, Germany) with the AMAXA Nucleofector II according to the manufacturer's instructions. Transfection efficacy for siRNA was determined 55–70% as determined with fluorescein conjugated control siRNA. Each transfected CD8^+^ T cells were rested for 6 hours and then cocultured with an equal number of CD14^+^ cells for 24 hours. After culture, CD107a and IFN-γ expression was analyzed in CD8^+^ T cells by flow cytometry.

### Western blot

SAP knockdown by siRNA was determined by western blot. After resting for 6 hours, transfected CD8^+^ T cells were collected and stored at −80°C until use. The cells were lysed in 5 mM Tris–HCl, pH 8.0, 1% Triton X-100 and a cocktail of protease inhibitors (Sigma). Protein concentration was determined using Quick Start Bradford Protein Assay (BioRad, Hercules, CA). From each protein sample, 10 µg was electrophoresed through a NuPAGE® 12% Bis–Tris gel (Invitrogen). The gel was transferred to a nitrocellurose membrane (Invitrogen). After blocking with 3% BSA in TBS, the membrane was probed with anti-SAP antibodies (Cell Signaling) and then probed with horseradish peroxidase-conjugated IgG (Cell Signaling). The membrane was visualized by chemiluminescence using SuperSignal® West Pico Chemiluminescent substrate (Thermo Scientific, Rockford, IL) and analyzed the profile on Kodak digital science™ 1D image analysis software (Kodak, Rochester, NY).

### Statistical analysis

Scatter plot and simple regression analysis were performed using Prism (GraphPad software, La Jolla, CA).

## References

[1] Verdonck K, Gonzalez E, Van Dooren S, Vandamme AM, Vanham G (2007). Human T-lymphotropic virus 1: recent knowledge about an ancient infection.. Lancet Infect Dis.

[2] Poiesz BJ, Ruscetti FW, Gazdar AF, Bunn PA, Minna JD (1980). Detection and isolation of type C retrovirus particles from fresh and cultured lymphocytes of a patient with cutaneous T-cell lymphoma.. Proc Natl Acad Sci U S A.

[3] Gessain A, Barin F, Vernant JC, Gout O, Maurs L (1985). Antibodies to human T-lymphotropic virus type-I in patients with tropical spastic paraparesis.. Lancet.

[4] Osame M, Usuku K, Izumo S, Ijichi N, Amitani H (1986). HTLV-I associated myelopathy, a new clinical entity.. Lancet.

[5] Umehara F, Izumo S, Nakagawa M, Ronquillo AT, Takahashi K (1993). Immunocytochemical analysis of the cellular infiltrate in the spinal cord lesions in HTLV-I-associated myelopathy.. J Neuropathol Exp Neurol.

[6] Levin MC, Lehky TJ, Flerlage AN, Katz D, Kingma DW (1997). Immunologic analysis of a spinal cord-biopsy specimen from a patient with human T-cell lymphotropic virus type I-associated neurologic disease.. N Engl J Med.

[7] Jacobson S, Shida H, McFarlin DE, Fauci AS, Koenig S (1990). Circulating CD8+ cytotoxic T lymphocytes specific for HTLV-I pX in patients with HTLV-I associated neurological disease.. Nature.

[8] Hanon E, Goon P, Taylor GP, Hasegawa H, Tanaka Y (2001). High production of interferon gamma but not interleukin-2 by human T-lymphotropic virus type I-infected peripheral blood mononuclear cells.. Blood.

[9] Kubota R, Kawanishi T, Matsubara H, Manns A, Jacobson S (2000). HTLV-I specific IFN-gamma+ CD8+ lymphocytes correlate with the proviral load in peripheral blood of infected individuals.. J Neuroimmunol.

[10] Nagai M, Yamano Y, Brennan MB, Mora CA, Jacobson S (2001). Increased HTLV-I proviral load and preferential expansion of HTLV-I Tax-specific CD8+ T cells in cerebrospinal fluid from patients with HAM/TSP.. Ann Neurol.

[11] Kannagi M, Shida H, Igarashi H, Kuruma K, Murai H (1992). Target epitope in the Tax protein of human T-cell leukemia virus type I recognized by class I major histocompatibility complex-restricted cytotoxic T cells.. J Virol.

[12] Greten TF, Slansky JE, Kubota R, Soldan SS, Jaffee EM (1998). Direct visualization of antigen-specific T cells: HTLV-1 Tax11-19- specific CD8(+) T cells are activated in peripheral blood and accumulate in cerebrospinal fluid from HAM/TSP patients.. Proc Natl Acad Sci U S A.

[13] Kubota R, Kawanishi T, Matsubara H, Manns A, Jacobson S (1998). Demonstration of human T lymphotropic virus type I (HTLV-I) tax-specific CD8+ lymphocytes directly in peripheral blood of HTLV-I-associated myelopathy/tropical spastic paraparesis patients by intracellular cytokine detection.. J Immunol.

[14] Jeffery KJ, Usuku K, Hall SE, Matsumoto W, Taylor GP (1999). HLA alleles determine human T-lymphotropic virus-I (HTLV-I) proviral load and the risk of HTLV-I-associated myelopathy.. Proc Natl Acad Sci U S A.

[15] Vine AM, Heaps AG, Kaftantzi L, Mosley A, Asquith B (2004). The role of CTLs in persistent viral infection: cytolytic gene expression in CD8+ lymphocytes distinguishes between individuals with a high or low proviral load of human T cell lymphotropic virus type 1.. J Immunol.

[16] Nagai M, Usuku K, Matsumoto W, Kodama D, Takenouchi N (1998). Analysis of HTLV-I proviral load in 202 HAM/TSP patients and 243 asymptomatic HTLV-I carriers: high proviral load strongly predisposes to HAM/TSP.. J Neurovirol.

[17] Yamano Y, Nagai M, Brennan M, Mora CA, Soldan SS (2002). Correlation of human T-cell lymphotropic virus type 1 (HTLV-1) mRNA with proviral DNA load, virus-specific CD8(+) T cells, and disease severity in HTLV-1-associated myelopathy (HAM/TSP).. Blood.

[18] Siekevitz M, Feinberg MB, Holbrook N, Wong-Staal F, Greene WC (1987). Activation of interleukin 2 and interleukin 2 receptor (Tac) promoter expression by the trans-activator (tat) gene product of human T-cell leukemia virus, type I.. Proc Natl Acad Sci U S A.

[19] Cross SL, Feinberg MB, Wolf JB, Holbrook NJ, Wong-Staal F (1987). Regulation of the human interleukin-2 receptor alpha chain promoter: activation of a nonfunctional promoter by the transactivator gene of HTLV-I.. Cell.

[20] Azimi N, Brown K, Bamford RN, Tagaya Y, Siebenlist U (1998). Human T cell lymphotropic virus type I Tax protein trans-activates interleukin 15 gene transcription through an NF-kappaB site.. Proc Natl Acad Sci U S A.

[21] Mariner JM, Lantz V, Waldmann TA, Azimi N (2001). Human T cell lymphotropic virus type I Tax activates IL-15R alpha gene expression through an NF-kappa B site.. J Immunol.

[22] Waldmann TA (2006). The biology of interleukin-2 and interleukin-15: implications for cancer therapy and vaccine design.. Nat Rev Immunol.

[23] Goon PK, Igakura T, Hanon E, Mosley AJ, Asquith B (2003). High circulating frequencies of tumor necrosis factor alpha- and interleukin-2-secreting human T-lymphotropic virus type 1 (HTLV-1)-specific CD4+ T cells in patients with HTLV-1-associated neurological disease.. J Virol.

[24] Enose-Akahata Y, Oh U, Grant C, Jacobson S (2008). Retrovirally induced CTL degranulation mediated by IL-15 expression and infection of mononuclear phagocytes in patients with HTLV-I-associated neurologic disease.. Blood.

[25] Hanon E, Stinchcombe JC, Saito M, Asquith BE, Taylor GP (2000). Fratricide among CD8(+) T lymphocytes naturally infected with human T cell lymphotropic virus type I.. Immunity.

[26] Ma CS, Nichols KE, Tangye SG (2007). Regulation of cellular and humoral immune responses by the SLAM and SAP families of molecules.. Annu Rev Immunol.

[27] Isomaki P, Aversa G, Cocks BG, Luukkainen R, Saario R (1997). Increased expression of signaling lymphocytic activation molecule in patients with rheumatoid arthritis and its role in the regulation of cytokine production in rheumatoid synovium.. J Immunol.

[28] Theil D, Farina C, Meinl E (2005). Differential expression of CD150 (SLAM) on monocytes and macrophages in chronic inflammatory contexts: abundant in Crohn's disease, but not in multiple sclerosis.. J Clin Pathol.

[29] Chan AY, Westcott JM, Mooney JM, Wakeland EK, Schatzle JD (2006). The role of SAP and the SLAM family in autoimmunity.. Curr Opin Immunol.

[30] Veillette A, Latour S (2003). The SLAM family of immune-cell receptors.. Curr Opin Immunol.

[31] Speiser DE, Colonna M, Ayyoub M, Cella M, Pittet MJ (2001). The activatory receptor 2B4 is expressed in vivo by human CD8+ effector alpha beta T cells.. J Immunol.

[32] Tangye SG, Phillips JH, Lanier LL, Nichols KE (2000). Functional requirement for SAP in 2B4-mediated activation of human natural killer cells as revealed by the X-linked lymphoproliferative syndrome.. J Immunol.

[33] Romero X, Benitez D, March S, Vilella R, Miralpeix M (2004). Differential expression of SAP and EAT-2-binding leukocyte cell-surface molecules CD84, CD150 (SLAM), CD229 (Ly9) and CD244 (2B4).. Tissue Antigens.

[34] Garni-Wagner BA, Purohit A, Mathew PA, Bennett M, Kumar V (1993). A novel function-associated molecule related to non-MHC-restricted cytotoxicity mediated by activated natural killer cells and T cells.. J Immunol.

[35] Valiante NM, Trinchieri G (1993). Identification of a novel signal transduction surface molecule on human cytotoxic lymphocytes.. J Exp Med.

[36] Nakajima H, Cella M, Langen H, Friedlein A, Colonna M (1999). Activating interactions in human NK cell recognition: the role of 2B4-CD48.. Eur J Immunol.

[37] Kubota K, Katoh H, Muguruma K, Koyama K (1999). Characterization of a surface membrane molecule expressed by natural killer cells in most inbred mouse strains: monoclonal antibody C9.1 identifies an allelic form of the 2B4 antigen.. Immunology.

[38] Peritt D, Sesok-Pizzini DA, Schretzenmair R, Macgregor RR, Valiante NM (1999). C1.7 antigen expression on CD8+ T cells is activation dependent: increased proportion of C1.7+CD8+ T cells in HIV-1-infected patients with progressing disease.. J Immunol.

[39] Blackburn SD, Shin H, Haining WN, Zou T, Workman CJ (2009). Coregulation of CD8+ T cell exhaustion by multiple inhibitory receptors during chronic viral infection.. Nat Immunol.

[40] Ostrowski SR, Ullum H, Pedersen BK, Gerstoft J, Katzenstein TL (2005). 2B4 expression on natural killer cells increases in HIV-1 infected patients followed prospectively during highly active antiretroviral therapy.. Clin Exp Immunol.

[41] Williams H, Macsween K, McAulay K, Higgins C, Harrison N (2004). Analysis of immune activation and clinical events in acute infectious mononucleosis.. J Infect Dis.

[42] Epling-Burnette PK, Painter JS, Rollison DE, Ku E, Vendron D (2007). Prevalence and clinical association of clonal T-cell expansions in Myelodysplastic Syndrome.. Leukemia.

[43] Kambayashi T, Assarsson E, Chambers BJ, Ljunggren HG (2001). Cutting edge: Regulation of CD8(+) T cell proliferation by 2B4/CD48 interactions.. J Immunol.

[44] Lee KM, Bhawan S, Majima T, Wei H, Nishimura MI (2003). Cutting edge: the NK cell receptor 2B4 augments antigen-specific T cell cytotoxicity through CD48 ligation on neighboring T cells.. J Immunol.

[45] Sayos J, Wu C, Morra M, Wang N, Zhang X (1998). The X-linked lymphoproliferative-disease gene product SAP regulates signals induced through the co-receptor SLAM.. Nature.

[46] Feldhahn N, Schwering I, Lee S, Wartenberg M, Klein F (2002). Silencing of B cell receptor signals in human naive B cells.. J Exp Med.

[47] Shlapatska LM, Mikhalap SV, Berdova AG, Zelensky OM, Yun TJ (2001). CD150 association with either the SH2-containing inositol phosphatase or the SH2-containing protein tyrosine phosphatase is regulated by the adaptor protein SH2D1A.. J Immunol.

[48] Chen R, Relouzat F, Roncagalli R, Aoukaty A, Tan R (2004). Molecular dissection of 2B4 signaling: implications for signal transduction by SLAM-related receptors.. Mol Cell Biol.

[49] Engel P, Eck MJ, Terhorst C (2003). The SAP and SLAM families in immune responses and X-linked lymphoproliferative disease.. Nat Rev Immunol.

[50] Itoyama Y, Minato S, Kira J, Goto I, Sato H (1988). Spontaneous proliferation of peripheral blood lymphocytes increased in patients with HTLV-I-associated myelopathy.. Neurology.

[51] Ward J, Bonaparte M, Sacks J, Guterman J, Fogli M (2007). HIV modulates the expression of ligands important in triggering natural killer cell cytotoxic responses on infected primary T-cell blasts.. Blood.

[52] Thorley-Lawson DA, Schooley RT, Bhan AK, Nadler LM (1982). Epstein-Barr virus superinduces a new human B cell differentiation antigen (B-LAST 1) expressed on transformed lymphoblasts.. Cell.

[53] Hanon E, Hall S, Taylor GP, Saito M, Davis R (2000). Abundant tax protein expression in CD4+ T cells infected with human T-cell lymphotropic virus type I (HTLV-I) is prevented by cytotoxic T lymphocytes.. Blood.

[54] Banerjee P, Feuer G, Barker E (2007). Human T-cell leukemia virus type 1 (HTLV-1) p12I down-modulates ICAM-1 and -2 and reduces adherence of natural killer cells, thereby protecting HTLV-1-infected primary CD4+ T cells from autologous natural killer cell-mediated cytotoxicity despite the reduction of major histocompatibility complex class I molecules on infected cells.. J Virol.

[55] Grakoui A, Bromley SK, Sumen C, Davis MM, Shaw AS (1999). The immunological synapse: a molecular machine controlling T cell activation.. Science.

[56] Sayos J, Nguyen KB, Wu C, Stepp SE, Howie D (2000). Potential pathways for regulation of NK and T cell responses: differential X-linked lymphoproliferative syndrome gene product SAP interactions with SLAM and 2B4.. Int Immunol.

[57] Roncagalli R, Taylor JE, Zhang S, Shi X, Chen R (2005). Negative regulation of natural killer cell function by EAT-2, a SAP-related adaptor.. Nat Immunol.

[58] Endt J, Eissmann P, Hoffmann SC, Meinke S, Giese T (2007). Modulation of 2B4 (CD244) activity and regulated SAP expression in human NK cells.. Eur J Immunol.

[59] Fehniger TA, Caligiuri MA (2001). Interleukin 15: biology and relevance to human disease.. Blood.

[60] Tomaru U, Yamano Y, Nagai M, Maric D, Kaumaya PT (2003). Detection of virus-specific T cells and CD8+ T-cell epitopes by acquisition of peptide-HLA-GFP complexes: analysis of T-cell phenotype and function in chronic viral infections.. Nat Med.

[61] Asquith B, Zhang Y, Mosley AJ, de Lara CM, Wallace DL (2007). In vivo T lymphocyte dynamics in humans and the impact of human T-lymphotropic virus 1 infection.. Proc Natl Acad Sci U S A.

[62] Benoit L, Wang X, Pabst HF, Dutz J, Tan R (2000). Defective NK cell activation in X-linked lymphoproliferative disease.. J Immunol.

[63] Sharifi R, Sinclair JC, Gilmour KC, Arkwright PD, Kinnon C (2004). SAP mediates specific cytotoxic T-cell functions in X-linked lymphoproliferative disease.. Blood.

[64] Dupre L, Andolfi G, Tangye SG, Clementi R, Locatelli F (2005). SAP controls the cytolytic activity of CD8+ T cells against EBV-infected cells.. Blood.

[65] Nagy N, Cerboni C, Mattsson K, Maeda A, Gogolak P (2000). SH2D1A and SLAM protein expression in human lymphocytes and derived cell lines.. Int J Cancer.

[66] Shinozaki K, Kanegane H, Matsukura H, Sumazaki R, Tsuchida M (2002). Activation-dependent T cell expression of the X-linked lymphoproliferative disease gene product SLAM-associated protein and its assessment for patient detection.. Int Immunol.

[67] Chlewicki LK, Velikovsky CA, Balakrishnan V, Mariuzza RA, Kumar V (2008). Molecular basis of the dual functions of 2B4 (CD244).. J Immunol.

[68] Oh U, Yamano Y, Mora CA, Ohayon J, Bagnato F (2005). Interferon-beta1a therapy in human T-lymphotropic virus type I-associated neurologic disease.. Ann Neurol.

